# The Influence of Metabolic Factors for Nonalcoholic Fatty Liver Disease in Women

**DOI:** 10.1155/2015/131528

**Published:** 2015-04-20

**Authors:** Goh Eun Chung, Jeong Yoon Yim, Donghee Kim, Seon Hee Lim, Jong In Yang, Young Sun Kim, Sun Young Yang, Min-Sun Kwak, Joo Sung Kim, Sang-Heon Cho

**Affiliations:** Department of Internal Medicine, Healthcare Research Institute, Gangnam Healthcare Center, Seoul National University Hospital, Seoul, Republic of Korea

## Abstract

*Background/Aims*. Women after menopause have increased insulin resistance and visceral fat, which may increase the prevalence of nonalcoholic fatty liver disease (NAFLD). However, the pathogenesis of NAFLD in women has not been clearly defined. In this study, we aimed to determine the risk factors for NAFLD in women. *Methods*. A retrospective cohort study was conducted. Women who underwent abdominal ultrasonography and blood sampling for routine health check-ups were recruited. *Results*. Among 1,423 subjects, 695 women (48.9%) were in a menopausal state. The prevalence of NAFLD was higher in postmenopausal women than in premenopausal women (27.2% versus 14.4%, *P* < 0.001). In premenopausal women, low HDL-cholesterol, central obesity, and homeostasis model assessment-estimated insulin resistance showed a significant association with the increased risk of NAFLD in multivariate analysis. In postmenopausal women, the presence of diabetes, triglyceridemia, and central obesity showed a significant association with the risk of NAFLD. The presence of menopause and hormone replacement therapy in postmenopausal women were not risk factors for NAFLD. *Conclusions*. Our findings showed different metabolic factors for NAFLD in pre- and postmenopausal women. However, the key issues are the same: central obesity and insulin resistance. These results reemphasize the importance of metabolic factors irrespective of menopausal status in the pathogenesis of NAFLD in women.

## 1. Introduction

Nonalcoholic fatty liver disease (NAFLD) is the leading cause of chronic liver disease, with increasing in prevalence up to 20–30% worldwide [1]. It is characterized by the accumulation of fat that is more than 5% of the hepatocytes in the liver [2], which encompasses various conditions ranging from simple steatosis, nonalcoholic steatohepatitis, to cirrhosis [3]. NAFLD is considered to be a hepatic manifestation of metabolic syndrome (MS), because the central pathogenesis of NAFLD is insulin resistance [4]. NAFLD is closely linked to variable components of MS such as type 2 diabetes, dyslipidemia, and central obesity [5, 6].

Women after menopause have increased insulin resistance and visceral abdominal fat, which are risk factors for the development of NAFLD [7]. Previous studies have indicated that gender differences and menopausal status may influence the prevalence and severity of NAFLD, suggesting an association with hepatic steatosis [8–11]. A recent study including patients with histological diagnosis of nonalcoholic steatohepatitis found that postmenopausal women as well as men have an increased risk for advanced fibrosis compared to premenopausal women [11]. NAFLD is more prevalent in postmenopausal women and women with polycystic ovary syndrome than those in a premenopausal state, suggesting a protective role of estrogen against NAFLD [12]. In a double-blind, randomized controlled study, hormone replacement therapy was associated with liver enzyme levels in women with type 2 diabetes [13]. However, the pathogenesis of NAFLD in women has not been clearly defined, and there is not much information about NAFLD and menopause or hormone replacement therapy, especially in population based studies. Therefore, the aim of this study was to determine the risk factors for NAFLD in women.

## 2. Patients and Methods

### 2.1. Study Population

A retrospective cohort study was conducted to evaluate the risk factors for NAFLD in women. Women who underwent abdominal ultrasonography (US) and blood samplings at the Seoul National University Hospital Gangnam Healthcare Center, Seoul, Republic of Korea, for routine health check-ups in 2010 were recruited. Most of the study population paid voluntarily for their health check-ups, and some of them were supported by their employer. Among 1,720 subjects, 57 subjects were positive for hepatitis B virus, 22 were positive for hepatitis C virus, 90 had significant alcohol consumption (>20 g/day), 2 had a history of chronic liver disease, and 126 had a past medical history of cancer and these subjects were excluded from the study. Finally, 1,423 subjects met the inclusion criteria. This study was approved by the Institutional Review Board of the Seoul National University Hospital with a waiver of informed consent.

### 2.2. Clinical and Laboratory Assessments

Each subject completed a past medical history questionnaire including the status of menopause and history of hormone replacement therapy. Women were considered menopausal if menstruation periods had stopped over 12 consecutive months previously. All subjects participated in anthropometric assessment and laboratory and radiologic tests on the same day. Body weight and height were measured using a digital scale, and body mass index (BMI) was calculated by dividing weight (kg) by the square of height (m^2^). Waist circumference was measured at the midpoint between the lower costal margin and the anterior superior iliac crest by a well-trained person using a tape measure. Systolic blood pressure and diastolic blood pressure were measured twice, and the mean values were reported. The presence of hypertension was defined as having a systolic blood pressure ≥140 mmHg or diastolic blood pressure ≥90 mmHg or use of antihypertensive medication. Current smokers were defined as having smoked at least 1 cigarette/day during the previous year. Former smokers were defined as prior regular cigarette smoking [14].

The laboratory tests included serum alanine aminotransferase (ALT), aspartate aminotransferase (AST), gamma-glutamyl transpeptidase (GGT), total cholesterol, triglycerides, high density lipoprotein (HDL) cholesterol, low density lipoprotein (LDL) cholesterol fasting glucose, hepatitis B surface antigen, and antibody to hepatitis C virus. Blood samples were collected before 10:00 am after a 12 h overnight fast. All laboratory tests were carried out using standard laboratory methods. Homeostasis model assessment-estimated insulin resistance (HOMA) was used to evaluate insulin resistance and was obtained by multiplying the fasting insulin by the fasting glucose. The presence of diabetes mellitus was defined as either a fasting serum glucose ≥126 mg/dL or use of antidiabetic medication.

MS was diagnosed when three or more of the five components were present, that is, (1) central obesity (waist circumference as defined by the Regional Office for the Western Pacific Region of the World Health Organization criteria, >90 cm (men) or >80 cm (women)); (2) a triglyceride level ≥150 mg/dL; (3) HDL-C <40 mg/dL (men) or <50 mg/dL (women); (4) fasting glucose ≥100 mg/dL or treatment for diabetes; (5) arterial pressure ≥130/85 mmHg or treatment for hypertension [15].

### 2.3. NAFLD Assessments

NAFLD was defined as the presence of fatty liver disease as determined by US with absence of the following: (1) seropositivity for hepatitis B surface antigens or antibodies to hepatitis C virus, (2) excessive alcohol intake (>20 g/day), (3) other causes of liver disease, and (4) medications known to produce fatty liver disease.

US examination of the liver was performed by experienced radiologists who were unaware of the clinical information. The diagnosis of fatty liver was determined by ultrasonography (Acusion, Sequoia 512, Siemens, Mountain View, CA) using previously described standardized criteria [16].

### 2.4. Statistical Analysis

Comparisons of continuous variables between the two groups were performed with Student's* t*-test, and categorical variables were compared using a chi-square test or Fisher's exact test. Variables that were statistically significant by univariate analysis and known risk factors were added to a multiple logistic regression model to identify independent predictors of the presence of NAFLD. Statistical analysis was performed with SPSS 19.0 (SPSS Inc.; Chicago, IL, USA). *P* values <0.05 were considered statistically to be significant.

## 3. Results

### 3.1. Baseline Characteristics

A total of 1,423 subjects were analyzed. The anthropometric, clinical, and laboratory characteristics of the subjects are shown in Table 1. Mean age was 52.4 ± 9.4 years. NAFLD was found in 294 (20.7%) subjects. Among the total subjects, 695 women (48.9%) were in menopausal state. Postmenopausal women had an older age, higher BMI, larger waist circumference, and higher levels of AST, ALT, GGT, fasting glucose, triglycerides, LDL-cholesterol, and HOMA than premenopausal women (*P* < 0.001). The presence of hypertension, diabetes, and MS was also higher in postmenopausal women compared to premenopausal women. The prevalence of NAFLD was higher in postmenopausal women than in premenopausal women (27.2%* versus* 14.4%, *P* < 0.001, Table 1).

In postmenopausal women, subjects with NAFLD had an older age, higher BMI, larger waist circumference, higher levels of AST, ALT, GGT, fasting glucose, triglycerides, and HOMA, and lower HDL-cholesterol than those without NAFLD (*P* < 0.001). The presence of hypertension, diabetes, MS, and NAFLD was higher in subjects with NAFLD compared to those without NAFLD. The rate of hormone replacement therapy was lower in subjects with NALFD than those without NAFLD (Table 2).

In premenopausal women, subjects with NAFLD had an older age, higher BMI, larger waist circumference, higher levels of AST, ALT, GGT, fasting glucose, triglycerides, LDL-cholesterol, and HOMA, and lower HDL-cholesterol than those without NAFLD (*P* < 0.001). The presence of hypertension, diabetes, MS, and NAFLD was higher in subjects with NAFLD compared to those without NAFLD (Table 3).

### 3.2. Risk Factors for NALFD in Women

We performed a logistic regression analysis to determine the risk factors for NAFLD in women. Univariate analysis showed that a postmenopausal state increased the risk of NAFLD (odds ratio 2.02 (1.42–2.86), *P* < 0.001); however, the statistical significance disappeared after adjusting for other confounding factors. Multivariate analysis indicated that diabetes, triglycerides levels, central obesity, and ALT levels increased the risk of NAFLD (Table 4).

Next, we stratified the subjects according to menopausal state. In postmenopausal women, the presence of diabetes, triglycerides levels, and central obesity showed a significant association with risk for NAFLD; however, hormone replacement therapy was not a risk factor for the development of NAFLD (Table 5). In premenopausal women, low HDL-cholesterol, central obesity, and HOMA showed a significant association with increased risk for NAFLD after multivariate analysis (Table 6).

## 4. Discussion

The study identified different metabolic factors for NAFLD in women in relation to menopause status; diabetes, triglycerides, and central obesity were risk factors in postmenopausal women, while low HDL-cholesterol, central obesity, and HOMA were risk factors in those with premenopausal status. However, in both cases key issues are the same: central obesity and insulin resistance. These results suggested that there is a close link between NAFLD and components of MS. Interestingly, the presence of menopause showed no significant association with increased risk of NAFLD, and hormone replacement therapy showed no association with NAFLD in postmenopausal women.

The prevalence of NAFLD in the current study (20.7%) was slightly higher than the prevalence (10–15%) reported in previous studies of Asian women [10, 17, 18]. However, the prevalence of NAFLD in postmenopausal women in this study (27.2%) was at the average compared to those in previous studies (ranging from 15% to 57%) [12, 17, 19]. These variable results between different studies may be due to different populations heterogeneously selected and the contributing various factors to the development of NAFLD in postmenopausal women.

Metabolic syndrome is a cluster of metabolic factors that are related to increased cardiovascular risks. The associations between NAFLD and MS have been well established [4, 20]. Obesity, hyperlipidemia, and type 2 diabetes, which are components of MS, are frequently coexisting conditions in subjects with NAFLD [21]. The incidence of MS was higher in subjects with NAFLD than those without NAFLD (33.8%* versus* 10.6%) [22]. In a Brazilian study on postmenopausal women, more than 90% of the subjects with NAFLD had MS [19]. Consistent with previous results, more than 50% of postmenopausal women with NAFLD had MS and multiple components of MS showed an association with NAFLD in our study. Among the various components of MS, central obesity was revealed as a key risk factor of NAFLD in both pre- and postmenopausal women, suggesting a critical role in the pathogenesis of NAFLD. In agreement with our results, visceral fat accumulation showed a positive correlation with hepatic steatosis [23, 24].

The prevalence of NAFLD was higher in postmenopausal women than premenopausal women in this study. Previous studies indicated that the menopausal status may influence the prevalence of NAFLD, suggesting the association with hepatic steatosis [8–11]. There have been several studies that have investigated the influence of estrogen on NAFLD. An animal study using the aromatase-knockout mouse showed that hepatic steatosis developed in aromatase-deficient mice [25, 26], and estradiol replacement therapy reversed hepatic steatosis, suggesting a role for estrogen in maintaining lipid homeostasis in the liver [27]. Previous human studies regarding a potent antagonist of estrogen, tamoxifen, showed that tamoxifen induced and progressed steatohepatitis in the treatment of breast cancer [28, 29]. A randomized controlled trial with tamoxifen performed in Italy showed that tamoxifen was associated with an increased risk of development of nonalcoholic steatohepatitis only in overweight and obese women [30]. Although the protective effect of estrogen in hepatic steatosis or NASH is distinct, the presence of menopause and hormone replacement therapy in postmenopausal women were not risk factors for NAFLD in this study, while metabolic risk factors had significant associations with an increased risk of NAFLD. The lack of association between menopause and NAFLD might be explained by potential mechanisms like the following. (i) Postmenopausal women have deficiencies in estrogen and relative androgen excess, which might lead to redistribution of total body fat such as increase of visceral fat, which might cause the development of insulin resistance [31]. Estrogen deficiency by itself is not as important as the relationship between testosterone and estradiol and exogenous administration of androgens to women has been demonstrated to insulin resistance [31–33], and clinical trials of postmenopausal women have not demonstrated benefits of exogenous estradiol [34–36]. In the same context, increased prevalence of NAFLD in patients with polycystic ovary syndrome is explained by androgen excess. Central adiposity and insulin resistance are the main factors related to NAFLD in postmenopausal women. (ii) In addition, multiple confounders such as metabolic factors, inflammation, nutritional factors, and age might influence the development of NALFD in postmenopausal women [9]. In this study, metabolic factors were associated significantly with NAFLD rather than menopause status. (iii) Older women have decreased physical activity and greater adiposity [37, 38], which may influence the changes in body composition and metabolic profiles. Consistent to our results, Hamaguchi et al. [17] indicated that weight gain and MS were independent risk factors for increased risk of NAFLD in both pre- and postmenopausal women; however, a postmenopausal state and hormone replacement therapy had no significant association with risk for NAFLD. Codes et al. showed that there was no difference in the frequency or severity of steatosis in relation to hormone replacement therapy in women with chronic hepatitis C [39].

A strength of this study is the determination of different metabolic factors in women with pre- or postmenopausal state. In addition, the subjects in this study are regarded to be representative of the general population due to the nature of being recruited at a health check-up visit. However, there were several limitations in the study. First, the design of a cross-sectional study makes it difficult to evaluate the temporal association between NAFLD and metabolic risk factors. Second, we could not obtain liver histology results, the gold standard diagnosis for NAFLD. US can introduce false-negative results when fatty infiltration is below 30% [40]. However, it was impossible to perform an invasive test in an apparently healthy population, and US is used as the first-line method for clinical practical guidelines [41, 42]. Third, information on dietary components and tests for inflammation were unavailable.

In conclusion, our findings showed different metabolic factors for NAFLD in pre- and postmenopausal women. However, the key issues are the same: central obesity and insulin resistance. These results reemphasize the importance of metabolic factors irrespective of menopausal status in the pathogenesis of NAFLD in women.

## Figures and Tables

**Table 1:** Comparison of baseline characteristics in premenopausal women versus postmenopausal women.

	Premenopausal women	Postmenopausal women	*P *
Number of subjects	728	695	<0.001
Age, years	46.9 ± 8.3	58.2 ± 6.7	<0.001
Body mass index, kg/m^2^	21.6 ± 2.7	22.6 ± 2.8	<0.001
Waist circumference, cm	78.7 ± 6.9	82.5 ± 7.4	<0.001
AST, IU/L	19.6 ± 6.7	22.9 ± 7.1	<0.001
ALT, IU/L	17.2 ± 9.9	21.8 ± 12.3	<0.001
GGT, IU/L	19.3 ± 14.7	24.3 ± 20.0	<0.001
Fasting glucose, mg/dL	90.5 ± 10.7	94.6 ± 14.9	<0.001
Triglycerides, mg/dL	84.4 ± 56.4	99.7 ± 59.4	<0.001
HDL-cholesterol, mg/dL	58.0 ± 13.2	51.0 ± 10.7	0.03
LDL-cholesterol, mg/dL	117.7 ± 27.9	128.4 ± 30.6	<0.001
CRP, mg/dL	0.08 ± 0.2	0.11 ± 0.24	0.019
HOMA	1.4 ± 1.0	1.6 ± 1.3	0.013
Smoking, %	2.8	2.2	0.291
Regular exercise, %	38.2	19.6	<0.001
Hypertension, %	17.2	36.2	<0.001
Diabetes, %	12.1	23.7	<0.001
Metabolic syndrome, %	8.8	25.9	<0.001
NAFLD, %	14.4	27.2	<0.001

Data are presented as the mean ± SD.

*P* value by Student's *t*-test for continuous variables and chi-square test for categorical variables.

ALT: alanine aminotransferase; AST: aspartate aminotransferase; GGT: gamma-glutamyl transpeptidase; HDL: high density lipoprotein; LDL: low density lipoprotein; CRP: C-reactive protein; HOMA: homeostasis model assessment-estimated insulin resistance; NAFLD: nonalcoholic fatty liver disease.

**Table 2:** Comparison of baseline characteristics in postmenopausal women without NAFLD versus with NAFLD.

	Postmenopausal women without NAFLD	Postmenopausal women with NAFLD	*P *
Number of subjects	506	189	
Age, years	57.7 ± 6.6	59.6 ± 6.7	<0.001
Body mass index, kg/m^2^	21.9 ± 2.3	24.5 ± 2.9	<0.001
Waist circumference, cm	80.8 ± 6.8	86.9 ± 7.0	<0.001
AST, IU/L	22.3 ± 5.8	24.6 ± 9.4	<0.001
ALT, IU/L	19.3 ± 8.9	28.3 ± 17.0	<0.001
GGT, IU/L	21.9 ± 17.3	30.9 ± 24.8	<0.001
Fasting glucose, mg/dL	92.1 ± 11.0	101.5 ± 20.5	<0.001
Triglycerides, mg/dL	86.7 ± 46.0	134.4 ± 75.4	<0.001
HDL-cholesterol, mg/dL	60.7 ± 12.0	54.0 ± 10.3	<0.001
LDL-cholesterol, mg/dL	127.6 ± 30.3	130.4 ± 31.3	0.216
CRP, mg/dL	0.09 ± 0.2	0.16 ± 0.28	0.006
HOMA	1.4 ± 1.2	2.2 ± 1.4	<0.001
Smoking, %	2.2	2.2	0.616
Regular exercise, %	17.4	25.9	0.012
Hypertension, %	30.1	52.4	<0.001
Diabetes, %	16.7	42.3	<0.001
Metabolic syndrome, %	16.8	50.3	<0.001
Hormone therapy	10.9%	6.3%	0.045

Data are presented as the mean ± SD.

*P* value by Student's *t*-test for continuous variables and chi-square test for categorical variables.

ALT: alanine aminotransferase; AST: aspartate aminotransferase; GGT: gamma-glutamyl transpeptidase; HDL: high density lipoprotein; LDL: low density lipoprotein; CRP: C-reactive protein; HOMA: homeostasis model assessment-estimated insulin resistance; NAFLD: nonalcoholic fatty liver disease.

**Table 3:** Comparison of baseline characteristics in premenopausal women without NAFLD versus with NAFLD.

	Premenopausal women without NAFLD	Premenopausal women with NAFLD	*P *
Number of subjects	623	105	
Age, years	46.3 ± 8.0	50.8 ± 9.0	<0.001
Body mass index, kg/m^2^	21.1 ± 2.3	24.6 ± 3.3	<0.001
Waist circumference, cm	77.5 ± 6.2	85.6 ± 7.2	<0.001
AST, IU/L	19.1 ± 5.9	22.7 ± 9.7	<0.001
ALT, IU/L	19.3 ± 8.9	28.3 ± 17.0	<0.001
GGT, IU/L	18.0 ± 13.3	27.3 ± 19.4	<0.001
Fasting glucose, mg/dL	89.1 ± 8.5	98.9 ± 17.0	<0.001
Triglycerides, mg/dL	75.5 ± 39.6	137.2 ± 97.8	<0.001
HDL-cholesterol, mg/dL	61.7 ± 12.3	52.0 ± 10.1	<0.001
LDL-cholesterol, mg/dL	115.7 ± 26.9	129.6 ± 30.7	<0.001
CRP, mg/dL	0.07 ± 0.2	0.13 ± 0.1	0.012
HOMA	1.2 ± 0.6	2.5 ± 1.8	<0.001
Smoking, %	2.9	1.9	0.742
Regular exercise, %	34.2	46.7	0.103
Hypertension, %	14.0	35.2	<0.001
Diabetes, %	8.5	33.3	<0.001
Metabolic syndrome, %	5.5	28.6	<0.001

Data are presented as the mean ± SD.

*P* value by Student's *t*-test for continuous variables and chi-square test for categorical variables.

ALT: alanine aminotransferase; AST: aspartate aminotransferase; GGT: gamma-glutamyl transpeptidase; HDL: high density lipoprotein; LDL: low density lipoprotein; CRP: C-reactive protein; HOMA: homeostasis model assessment-estimated insulin resistance; NAFLD: nonalcoholic fatty liver disease.

**Table 4:** Unadjusted and adjusted analyses for the risk of NAFLD in all subjects.

	Unadjusted OR (95% CI)	*P *	Adjusted OR (95% CI)	*P *
Age	1.05 (1.03–1.07)	<0.001	1.00 (0.97–1.03)	0.989
Postmenopause	2.02 (1.42–2.86)	<0.001	1.07 (0.64–1.79)	0.803
Hypertension	2.97 (2.09–4.21)	<0.001	1.52 (0.96–2.38)	0.073
Diabetes	4.52 (3.08–6.64)	<0.001	2.35 (1.40–3.95)	0.001
Triglyceridemia	6.47 (4.12–10.18)	<0.001	3.22 (1.81–5.72)	<0.001
HDL	0.33 (0.23–0.48)	<0.001	0.60 (0.38–0.97)	0.036
Central obesity	6.45 (4.48–9.27)	<0.001	3.67 (2.36–5.70)	<0.001
Smoking	0.93 (0.34–2.53)	0.891	0.75 (0.23–2.46)	0.628
Exercise	1.43 (1.00–2.05)	0.052	1.56 (0.99–2.47)	0.057
ALT	1.08 (1.06–1.10)	<0.001	1.07 (1.04–1.09)	<0.001
GGT	1.02 (1.01–1.03)	<0.001	1.00 (0.99–1.01)	0.353
HOMA	2.33 (1.91–2.54)	<0.001	1.23 (0.97–1.56)	0.093

NAFLD: nonalcoholic fatty liver disease; ALT: alanine aminotransferase; GGT: gamma-glutamyl transpeptidase; OR: odds ratio; CI: confidence interval; HDL: high density lipoprotein; HOMA: homeostasis model assessment-estimated insulin resistance.

**Table 5:** Unadjusted and adjusted analyses for the risk of NAFLD in postmenopausal women.

	Unadjusted OR (95% CI)	*P *	Adjusted OR (95% CI)	*P *
Age	1.03 (0.99–1.06)	0.124	0.97 (0.93–1.01)	0.179
Hypertension	2.68 (1.73–4.17)	<0.001	1.50 (0.86–2.61)	0.152
Diabetes	3.57 (2.22–5.74)	<0.001	2.38 (1.30–4.37)	0.005
Triglyceridemia	5.57 (3.09–10.03)	<0.001	4.51 (2.16–9.43)	<0.001
HDL	0.46 (0.29–0.75)	0.002	0.85 (0.46–1.57)	0.592
Central obesity	4.91 (3.10–7.76)	<0.001	4.17 (2.40–7.23)	<0.001
Hormone therapy	0.03 (0.13–0.88)	0.026	0.47 (0.15–1.44)	0.185
Smoking	0.78 (0.21–2.89)	0.713	0.56 (0.13–2.45)	0.439
Exercise	1.59 (0.96–2.62)	0.072	1.35 (0.74–2.48)	0.332
ALT	1.07 (1.04–1.10)	<0.001	1.07 (1.04–1.10)	<0.001
GGT	1.01 (1.00–1.02)	0.022	0.99 (0.98–1.00)	0.122
HOMA	1.72 (1.37–2.16)	<0.001	1.02 (0.84–1.24)	0.838

NAFLD: nonalcoholic fatty liver disease; ALT: alanine aminotransferase; GGT: gamma-glutamyl transpeptidase; OR: odds ratio; CI: confidence interval; HDL: high density lipoprotein; HOMA: homeostasis model assessment-estimated insulin resistance.

**Table 6:** Unadjusted and adjusted analyses for the risk of NAFLD in premenopausal women.

	Unadjusted OR (95% CI)	*P *	Adjusted OR (95% CI)	*P *
Age	1.03 (0.99–1.06)	0.124	1.04 (0.99–1.10)	0.121
Hypertension	2.68 (1.73–4.17)	<0.001	1.57 (0.69–3.55)	0.283
Diabetes	3.57 (2.22–5.74)	<0.001	2.07 (0.77–5.53)	0.149
Triglyceridemia	5.57 (3.09–10.03)	<0.001	1.75 (0.62–5.00)	0.293
HDL	0.46 (0.29–0.75)	0.002	0.36 (0.16–0.83)	0.016
Central obesity	4.91 (3.10–7.76)	<0.001	2.79 (1.25–6.24)	0.012
Smoking	0.78 (0.21–2.89)	0.713	0.70 (0.08–6.14)	0.744
Exercise	1.59 (0.96–2.62)	0.072	1.70 (0.79–3.66)	0.176
ALT	1.07 (1.04–1.10)	<0.001	1.06 (1.02–1.10)	0.003
GGT	1.01 (1.00–1.02)	0.022	1.01 (0.99–1.03)	0.475
HOMA	1.72 (1.37–2.16)	<0.001	2.46 (1.51–4.00)	<0.001

NAFLD: nonalcoholic fatty liver disease; ALT: alanine aminotransferase; GGT: gamma-glutamyl transpeptidase; OR: odds ratio; CI: confidence interval; HDL: high density lipoprotein; HOMA: homeostasis model assessment-estimated insulin resistance.

## Abbreviations

ALT: Alanine aminotransferase
BMI: Body mass index
HDL: High density lipoprotein
MS: Metabolic syndrome
NAFLD: Nonalcoholic fatty liver disease
AST: Aspartate aminotransferase
GGT: Gamma-glutamyl transpeptidase
HOMA: Homeostasis model assessment-estimated insulin resistance
US: Ultrasonography
LDL: Low density lipoprotein.
